# Using Ecological Momentary Assessment, Geolocation Tracking, and Neuroimaging to Assess Effects of Tobacco Retail Exposure on Smoking Behavior: Protocol for the GeoSmoking Study

**DOI:** 10.2196/89627

**Published:** 2026-07-10

**Authors:** Nicole Cooper, Benjamin Muzekari, Anthony Resnick, Alexandra M Paul, Omaya Torres-Grillo, Farah Sayed, Elizabeth Beard, Melis E Cakar, Jose Carreras-Tartak, Susan Hao, Bradley Mattan, Mary Andrews, Darin Johnson, Christian Benitez, Emily Zhou, Christin Scholz, Ian Barnett, Michael Fichman, Lisa Henriksen, Thomas R Kirchner, David M Lydon-Staley, Andrew A Strasser, Emily B Falk

**Affiliations:** 1 Annenberg School for Communication, University of Pennsylvania, 3620 Walnut St, Philadelphia, PA, 19104, United States, 1 (215) 573-9901; 2 College of Medicine, University of Cincinnati, Cincinnati, OH, United States; 3 Wharton AI and Analytics Initiative, University of Pennsylvania, Philadelphia, PA, United States; 4 Neuroscience Interdepartmental Program, University of California, Los Angeles, Los Angeles, CA, United States; 5 Alan Alda Center for Communicating Science, Stony Brook University, Stony Brook, NY, United States; 6 School of Communication and Journalism, Stony Brook University, Stony Brook, NY, United States; 7 School of Journalism and Mass Communications, University of South Carolina, Columbia, SC, United States; 8 Department of Psychology, School of Arts and Sciences, University of Pennsylvania, Philadelphia, PA, United States; 9 Department of Geography, University of California, Santa Barbara, Santa Barbara, CA, United States; 10 Amsterdam School of Communication Research, University of Amsterdam, Amsterdam, North Holland, The Netherlands; 11 Department of Biostatistics, University of Pennsylvania, Philadelphia, PA, United States; 12 Department of City and Regional Planning, Weitzman School of Design, University of Pennsylvania, Philadelphia, PA, United States; 13 Stanford Prevention Research Center, School of Medicine, Stanford University, Palo Alto, CA, United States; 14 Department of Social and Behavioral Sciences, School of Global Public Health, New York University, New York City, NY, United States; 15 Tandon School of Engineering, New York University, New York, NY, United States; 16 Department of Bioengineering, School of Engineering and Applied Sciences, University of Pennsylvania, Philadelphia, PA, United States; 17 Leonard Davis Institute of Health Economics, University of Pennsylvania, Philadelphia, PA, United States; 18 Center for Interdisciplinary Research on Nicotine Addiction, Department of Psychiatry, University of Pennsylvania, Philadelphia, PA, United States; 19 Penn Medicine Abramson Cancer Center, University of Pennsylvania, Philadelphia, PA, United States; 20 Wharton Marketing Department, University of Pennsylvania, Philadelphia, PA, United States; 21 Wharton Operations, Information and Decisions Department, University of Pennsylvania, Philadelphia, PA, United States; 22 Annenberg Public Policy Center, University of Pennsylvania, Philadelphia, PA, United States

**Keywords:** ecological momentary assessment, environmental exposure, geolocation, health, smartphone, substance use, tobacco control, neural cue reactivity

## Abstract

**Background:**

Cigarettes are a global public health concern, as cigarette smoking is the leading cause of death in the United States and throughout most high-income countries. Exposure to tobacco retail has been linked to adverse smoking outcomes, but research using naturalistic and causal approaches to quantify these effects in the real world remains relatively sparse. To address these gaps, this study used geolocation tracking, ecological momentary assessment, and neuroimaging to assess smoking outcomes in daily life and conducted a randomized controlled trial focused on the effects of exposure to tobacco retail.

**Objective:**

The GeoSmoking study aimed to evaluate (1) within-person associations between real-world tobacco retail exposure and cigarette craving and smoking, (2) causal effects of real-world tobacco retail exposure, and (3) neural cue reactivity as a mechanism for real-world tobacco retail effects. This paper describes the implemented protocol, including study status and flow reporting.

**Methods:**

In a 2-week baseline period, the study collected reports of craving and smoking multiple times per day using ecological momentary assessment, in addition to other measures. Simultaneously, geolocation tracking was used to quantify tobacco retail exposure through the creation of a tobacco retail database across 3 US states (Pennsylvania, New Jersey, and Delaware). A 4-week intervention period followed, in which participants were randomly assigned to make a purchase at either a nontobacco retail store 5 days per week (nontobacco retail condition) or a tobacco retail store 5 days per week (tobacco retail condition), or follow their normal routines (control condition). An optional functional magnetic resonance imaging (fMRI) session concluded the study. Individuals participated remotely, unless they opted into the fMRI session, which was completed at the University of Pennsylvania.

**Results:**

The GeoSmoking study was approved by the Institutional Review Board at the University of Pennsylvania. Data collection started on May 25, 2022, and ended on June 10, 2024. In total, 310 participants were enrolled, 282 participants completed the baseline phase, 244 participants completed the intervention phase, and 24 participants completed the optional fMRI scan.

**Conclusions:**

This study protocol was implemented successfully. Findings from planned analyses will quantify the strength of relationships between naturalistic exposure to tobacco retail, craving, smoking, and a range of other outcomes. These findings have significant implications for our understanding of health behaviors and outcomes, as well as policy.

## Introduction

### Cigarette Smoking and Tobacco Retail

Cigarette smoking is the leading cause of preventable death and illness in the United States and throughout most high-income countries [1]. Smoking increases the odds of developing the most common cancers and increases the risk for other leading causes of death, accounting for 1 in 5 deaths in the United States and more than 7 million deaths worldwide annually [2,3]. The tobacco industry spends US $8.4 billion—or 96.8% of its yearly marketing budget—in the retail environment [4]. Tobacco advertising and products are featured prominently in “power walls” near or behind cash registers in retail outlets like convenience stores and gas stations [5-7], such that all customers are exposed to this marketing. Tobacco advertising is present in approximately 92% of convenience stores, with 93% of tobacco displays being in the counter zone [8,9]. Tobacco advertising is also often placed on the exterior of storefronts, particularly in urban communities [10-12]; upward of approximately 70% of some types of stores (eg, convenience stores and gas stations) display outdoor tobacco advertising [12]. The majority of youth in the United States report noticing tobacco product advertising almost every time they enter a convenience store [8,9]. Such widespread and frequent exposure to tobacco retail marketing is of great interest to tobacco control and cancer prevention research worldwide.

An extensive scientific literature has identified negative effects of tobacco retail exposure on smoking outcomes [3,13,14]. However, there is little research using naturalistic and time-sensitive approaches to assessing exposure to tobacco retail within individuals, across time in their daily lives, evaluating real-world causal effects of changes in exposure to tobacco retail on smoking outcomes, and examining neural cue reactivity to marketing cues as a mechanistic explanation for the effects of tobacco retail marketing on behavior. Each of these topics has important implications for understanding the effects that tobacco retail exposure has on smoking. Therefore, the GeoSmoking study was designed to address these gaps through an innovative and multimodal protocol that assesses individuals’ day-to-day experiences in their natural environments, leveraging insights and methods from across the social sciences. This implemented research protocol paper describes the design rationale for the GeoSmoking study and details of the protocol, as well as participant flow and retention, and descriptive information about the study sample.

### Assessing Exposure to Tobacco Retail

A core focus of the GeoSmoking study was to assess individuals’ exposure to tobacco retail, including the built and marketing environments. Prior work suggests detrimental associations between such exposures and smoking outcomes, including increased craving, purchase urges, impulse purchases, and odds of failed cessation attempts [15-20]. However, there remains a substantial gap in understanding real-time, repeated, and naturalistic within-person associations between tobacco retail exposure and smoking outcomes in adults who smoke daily. To enhance ecological validity, some prior research has used place-based approaches in which a participant’s home address or neighborhood is used to approximate real-world exposure. These place-based estimates of exposure are then later correlated with health behaviors, such as smoking [21-28]. In this approach, people’s exposure is estimated based on static information (eg, home or school address) exclusively, and therefore, these studies are unable to evaluate exposures dynamically within individuals across time. Further, these limited exposure measures cannot capture people’s daily mobility outside of their home, such as traveling for work, errands, or recreation. More dynamic measures are needed to better capture these variations in order to assess individuals’ daily mobility and exposures to tobacco retail.

The GeoSmoking study used geolocation tracking, which allows objective measurement of individuals’ exposure to environmental risk factors in real time. The recent ubiquity of geolocation tracking built into smartphones provides the opportunity for incorporating this methodology into research designs with high accessibility and low participant burden. A pivotal study compared tobacco retail exposure estimates nationwide in the United States using neighborhood approximated exposure to real-time geolocation tracking and found that 55% of the variance accounted for by geolocation tracking was not accounted for using neighborhood approximated exposure [29]. Results suggest that approximating tobacco retail exposure using geolocation tracking may provide different, additional information relative to neighborhood-approximated exposure alone. Such methods allow researchers to measure environmental exposures more precisely, both geographically and temporally, as opposed to relying on a single proxy or self-reported exposure. Using geolocation tracking, exposure to tobacco retail has been associated with adolescents’ daily smoking [30,31], as well as with smoking cessation success in adults [32]. The GeoSmoking study expanded this methodology to track individuals over a relatively long period of time (6 weeks) and to test associations between exposure to tobacco retail and smoking outcomes in adults who are smoking as usual.

### Experimental Approaches to Investigating the Effects of Tobacco Retail Exposure

A second component of the GeoSmoking study was the use of experimental methods to explore the causal effects of exposure to tobacco retail. Leveraging geolocation tracking and geospatial information science is key to gaining real-world insights about tobacco retail exposure and smoking outcomes, but the approaches described above are inherently correlational. Thus, another key question is whether real-world tobacco retail exposure has causal impacts on craving and smoking. Recent work has used either online virtual stores or full-size convenience store replicas to test causal effects of exposure to retail and tobacco retail marketing. Such research demonstrates that removing retail marketing, or reducing its visibility, reduces purchase attempts [33], urges to smoke [33], and susceptibility to smoke among those who have never tried [34]. In an online experiment, individuals who currently smoked reported greater craving after viewing point-of-sale cigarette promotion cues compared to nonsmoking cues, which provided evidence that viewing images of tobacco marketing can increase craving [35].

Together, these experimental studies featured randomized and controlled designs in tightly structured environments, strengthening internal validity for the effects of retail exposure on smoking outcomes. However, there is no extant research that examines real-world causal effects across time and within individuals. Thus, an important question is whether real-world visits to tobacco retail stores have causal effects on craving and smoking in daily life, outside of highly controlled lab settings.

### Neural Mechanisms of Smoking Cue Reactivity

A final focus of the GeoSmoking study was the brain’s response to tobacco retail marketing. Theoretical models and empirical studies of addiction suggest that exposure to drug cues, such as images of drug paraphernalia, enhances craving and consumption in individuals who use drugs, including those who smoke cigarettes [36,37]. Researchers have investigated the brain mechanisms of these associations, which can inform intervention strategies, such as using functional magnetic resonance imaging (fMRI) neurofeedback, to decrease neural reactivity during exposure to cues that are known to elicit reward activation [38]. Laboratory work has shown increased neural activity in regions associated with drug cue-reactivity, as well as increased ratings of subjective craving, after exposure to smoking cues such as pictures of cigarettes [39]. Craving ratings scale positively with neural cue-reactivity [40-42], and both metrics predict smoking behaviors in a later ad-lib smoking session [43,44].

Neural cue reactivity effects have been generalized from completely standardized cues to more naturalistic pictures of personal smoking environments, such that exposure to photographs of places where participants often smoke (eg, a bus stop where an individual smokes their morning cigarette) evokes stronger neural and behavioral craving responses than exposure to generic photographs (eg, an unfamiliar bus stop) [44-46]. This highlights that smoking can be interconnected with an individual’s life and what they see and experience in their environments. Recent work assessed neural responses to photographs of familiar or unfamiliar tobacco retail stores and found higher responses in regions related to self-relevance and smoking motivation [47]. However, this study did not assess self-reported craving in response to these photographs, and only the outside of retail stores was displayed, leaving out images of internal marketing, including power walls. Such brain responses have also not been linked to real-world craving and smoking in response to tobacco retail encountered in daily life.

Thus, laboratory experiments implicate neural smoking cue reactivity and cigarette craving as mechanisms linking exposure to smoking cues and behavior, but significant questions remain about whether viewing tobacco marketing elicits self-reported craving as well as activation in the same brain regions as standardized, proximal smoking cues like pictures of cigarettes, and how such reactivity relates to real-world vulnerability to tobacco retail marketing. Knowledge of these mechanisms can help inform intervention approaches and provide opportunities such as using the brain as a predictor of real-world effects.

### The GeoSmoking Study

The GeoSmoking study was designed to address each of these 3 main gaps in the literature. First, the study used a dynamic, real-world assessment of exposure to tobacco retail. Recruitment targeted adults who smoked cigarettes daily and who lived in 1 of 3 US states (Pennsylvania [PA], Delaware [DE], and New Jersey [NJ]) to participate in a remote, longitudinal study. In a 2-week baseline period, ecological momentary assessment (EMA) was used to capture reports of craving and smoking throughout the day, and geolocation tracking to assess real-world mobility. The study team curated a database of all tobacco retail stores in the 3 states and used these data to assess participants’ exposure to tobacco retail in the real world. This allowed examination of within-person associations between real-world tobacco retail exposure and cigarette craving and smoking.

In addition, the study team designed an experiment to intervene on participants’ real-world exposure to tobacco retail. The baseline period was followed by a month-long experiment in which participants’ levels of tobacco retail exposure were manipulated by assigning them to make a purchase at a store from a randomly assigned retail chain 5 days per week, while geolocation tracking and EMA continued. Participants were assigned to enter a conveniently located branch of one of two widely known retail chains: a convenience store that sells tobacco (tobacco retail condition) or a pharmacy chain that does not sell tobacco (nontobacco retail condition). A third control condition instructed participants to follow their normal routine. The goal of this manipulation was to examine the causal effects of real-world tobacco retail exposure on cigarette craving and smoking.

Finally, the study team designed an fMRI task to examine the neural mechanisms of cue reactivity. The study concluded with an optional, in-person fMRI visit in which participants completed image rating tasks, which included tobacco marketing cues. The goal of the fMRI task was to examine neural cue reactivity as an explanatory mechanism for how real-world marketing affects craving and smoking.

The GeoSmoking study had three core aims:

1. Examine within-person associations between real-world tobacco retail exposure and cigarette craving and smoking
2. Examine the causal effects of real-world tobacco retail exposure on cigarette craving and smoking
3. Examine neural cue reactivity as an explanatory mechanism for how exposure to real-world marketing relates to craving and smoking

The study’s innovative approach and its resulting findings can inform policy changes aimed at reducing smoking, as well as the design of health behavior change interventions. Data collection for the study is complete. The next sections describe the study design, research protocol, and data collection results.

## Methods

### Study Summary

**Figure 1.**
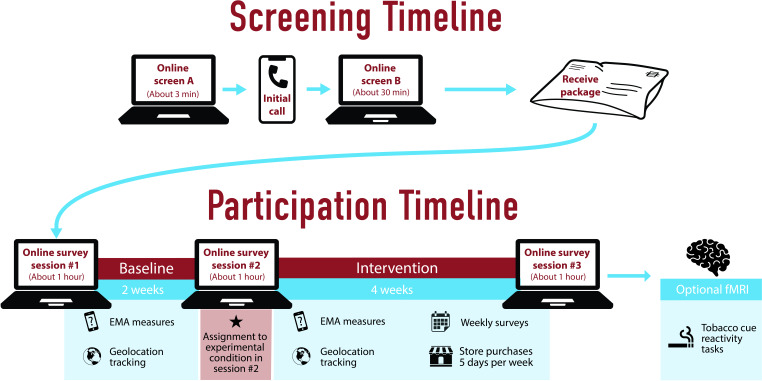
Participant screening and participation timeline. The screening steps (top row) were all completed before participants consented to participate in the main study at the start of Online Survey Session 1, after which eligible participants continued to the main study tasks (bottom row). EMA: ecological momentary assessment; fMRI: functional magnetic resonance imaging.

The GeoSmoking study was conducted using remote sessions through a protocol approved by the University of Pennsylvania’s Institutional Review Board (IRB). Data collection started on May 25, 2022, and ended on June 10, 2024. Adults who smoked cigarettes daily were recruited across PA, DE, and NJ to complete a multipart, remote study. As shown in Figure 1, participants completed the study over a minimum of 6 weeks, which included a screening period, a 2-week baseline period, a 4-week intervention period, and an optional on-campus fMRI session (full participation timeline described in detail in Study Timeline). In brief, during the 2-week baseline period, participants reported craving and smoking via EMA multiple times per day while their geolocation was tracked to calculate tobacco retail exposure. The following 4-week intervention period was designed to assess the causal effects of exposure to tobacco retail. Participants were assigned to enter a tobacco retail store or a nontobacco retail store 5 times per week, or to follow their normal routines, while EMA and geolocation continued to be collected. Following the intervention period, eligible participants could opt into an fMRI session conducted at the University of Pennsylvania, designed to investigate neural reactivity to tobacco marketing cues.

### Recruitment

Recruitment materials for this study were primarily distributed by BuildClinical, a health technology company supporting recruitment for clinical trials. BuildClinical used study advertisements to engage participants on digital platforms such as Facebook, Google, and WebMD. All recruitment materials included links (eg, URLs and QR codes) directing potential participants to the study landing page [48], which then directed them to complete the initial screening survey (Online Screen A) on the BuildClinical platform. Information collected via BuildClinical was transferred securely to REDCap (Research Electronic Data Capture), an online platform for survey and database management. Eligible participants were automatically identified through a built-in reporting feature on the REDCap database. These eligible participants were notified via text and email that they may be eligible to enroll in the study and were invited to schedule an initial phone screening call with study staff.

Some participants requested referral links for other potential participants. In these situations, participants were sent a direct link to the screening survey in REDCap. Participants were asked not to talk about the details of the study with other potential participants until after they both completed their participation.

The study team contacted active and completed participants who were potentially eligible for the optional fMRI session to screen them for interest in and eligibility for the scan session.

### Screening Period

#### Eligibility Criteria

Eligible criteria verified during the screening period were as follows: (1) ages 21‐65 years; (2) smoked at least 5 cigarettes a day for the past 6 months; (3) owned an iPhone or Android smartphone that could be used on a daily basis; (4) residents of PA, NJ, or DE; (5) fluent in English; and (6) fully vaccinated against COVID-19. The latter was required because the COVID-19 pandemic was classified as a federal public health emergency when the study began. Therefore, extra precautions were implemented to reduce study risks for participants who were asked to visit retail stores. Individuals were excluded during the screening process if they did not meet the eligibility criteria above or for meeting one or more of the following exclusion criteria: concurrent enrollment or plans to enroll in a smoking cessation program within 3 months of completing the screening survey; plans to use nicotine substitutes or smoking cessation treatments within 3 months of completing the screening survey; demonstration of urine cotinine concentration below 200 ng/mL (≥200 ng/mL confirmed smoking status); pregnancy; refusal to install Google Maps or LifeData apps on their mobile phone; if their phone’s functionality did not allow for adequate completion of study tasks; inability or refusal to upload Google Timeline data after receiving instructions and any additional guidance; or any planned, extended trips outside of PA, NJ, and DE during the study phase (participants were also given the option to postpone enrollment). The main study goals did not involve differences by sex, gender, race, or ethnicity, and, therefore, there were no recruitment restrictions by these variables. Any potential participant who expressed interest and was eligible to participate was able to enroll in the study from the study outset.

#### Screening Timeline

##### Step 1: Online Screen A

Those who expressed interest in the study by reacting to the recruitment efforts were directed to an online screening survey (Online Screen A), where they answered questions to determine their eligibility for the study and provided their contact information. Online Screen A also asked about the participants’ interest in receiving information about opportunities to participate in other remote and/or in-person studies. The screening survey was administered online via BuildClinical or REDCap and took approximately 3 minutes to complete. Ineligible individuals were informed via email. Survey respondents who were potentially eligible to participate were emailed and texted a link to an appointment booking system to sign up for a time for an initial screening phone call.

##### Step 2: Initial Call

Before the initial call, potentially eligible participants were emailed a copy of the consent form and study timeline for review. During the initial call, participants were given more details about the study, including the risks and benefits of participation, and were given the opportunity to ask questions. Eligibility criteria were also confirmed on this call as needed. The researcher conducting the call entered participant responses into REDCap. At the end of the initial call, potential participants who were still eligible and interested were invited via email to complete Online Screen B at a time of their choosing within 1 month of the initial call.

##### Step 3: Online Screen B

At the beginning of Online Screen B, participants signed an electronic consent form for additional eligibility screening. Potential participants uploaded a photograph of their COVID-19 vaccination card to verify that they were fully vaccinated against COVID-19 (according to standards at the time of their consent). Participants also supplied their mailing address for delivery of a package with study supplies. The online instructions then demonstrated how to download and/or set up Google Maps with the correct settings for location tracking on their phone, followed by guidance on how to export and upload their Google Timeline data (which contains their geolocation history). Separate detailed instructions were written and customized for both iPhone and Android operating systems. Study staff provided the option of a guided phone call to assist with these steps, and potential participants could request technical help at any time. Each potential participant needed to meet the following requirements: (1) they lived in the study area (PA, NJ, or DE), according to their provided shipping address and uploaded location history, and (2) they were fully vaccinated against COVID-19, according to their vaccination card and standards at the time of their consent. Those who were unable or unwilling to complete any component of Online Screen B, lived outside of the specified study area, or were not fully vaccinated were ineligible for the study. If an individual was found to be ineligible at this point, the team informed them of their status, deleted their geolocation data, and provided them with instructions for turning off location tracking through Google Timeline and uninstalling Google Maps (if desired).

##### Step 4: Study Materials

A box of study materials was mailed to each potential participant, including a sterile, sealed sample collection cup, two urine cotinine tests (one for backup, in case of inconclusive result or error), a Greenphire Clincard for receipt of study payments, and KN95/KF94 or N95 masks (supplied to reduce risk of contracting COVID-19 during study-assigned visits to nontobacco retail or tobacco retail stores). After receiving the study materials, potential participants were able to begin Online Session 1.

### Main Study Period

#### Postenrollment Exclusion Criteria

##### Baseline Exclusions

If a participant’s response rate to the EMA prompts was below 75% for the baseline period, they were excluded from participating in the intervention period and were not assigned to an experimental condition. Participants were also excluded for noncompliance at the end of the baseline period if their geolocation tracking was not turned on during the baseline period (based on the location history file they provided). Participants were able to pause their participation as needed (eg, due to significant illness or disruptive life events) at any point during the study.

From the study start date (May 25, 2022) until October 13, 2022, participants were excluded during the intervention period if they stated that they would not make store visits, if it became clear that they could not complete 20 visits before the end of the intervention period, or if their EMA response rate for the intervention period could not reach 75%. This protocol was implemented for the first 65 participants. On October 13, 2022, the study team moved to an intent-to-treat approach and did not exclude participants due to noncompliance once they began the intervention period. This approach was implemented to preserve randomization and minimize biases. Beginning on October 6, 2023, in order to increase protocol compliance, participants were asked at the completion of the baseline period if they would still be able to complete store visits, should they be assigned to either the nontobacco retail or tobacco retail store conditions. This was asked before participants knew their assigned condition. If participants indicated that they were no longer able to complete the store visits, they were not randomized into an experimental condition, excluded from further participation, and asked to upload one final location history file. Upon location file upload, these participants were then paid for the tasks they completed up to the point of their exclusion.

##### fMRI Session

Participants who completed the baseline and intervention periods were eligible to participate in the optional fMRI component unless they met one or more of the following exclusion criteria: demonstration of urine cotinine concentration below 200 ng/mL (≥200 ng/mL confirmed smoking status) at scanning session; currently or recently (within the last 5 years) received medical treatment for a substance use disorder (treatment of substance use disorders that occurred greater than 5 years prior to study participation was acceptable if participants were in stable condition); report consuming any of the following drugs within the past 2 weeks or indicate plans to do so within the coming 6 weeks during the fMRI screening call: benzodiazepines, amphetamines, methamphetamines, cocaine, methylenedioxymethamphetamine, methadone, barbiturates, phencyclidine, heroin, oxycodone, opiates (eg, morphine and heroin), buprenorphine; test positive for any of the above drugs at the scan appointment; schizophrenia or psychosis, regardless of treatment status; history of stroke or other neurological disorder likely to affect cognition; psychiatric hospitalization within the past year; propensity to experience claustrophobia; ferromagnetic metal in the body, including objects that might set off a metal detector and were not preapproved as safe by trained MRI technicians; metal in the body of an unverifiable origin; nonremovable piercings; nonremovable retainers or other dental work not compatible with fMRI; any orthopedic implant above the neck; participants whose weight exceeded 350 pounds, due to constraints of the fMRI scanner; any medical condition or concomitant medication that could compromise participant safety or treatment, as determined by the principal investigator and/or study physician; unable to schedule a scan within 6 months after completing the third online session.

### Main Study Timeline

#### Step 1: Online Session 1

In this session, participants read through the consent form and provided electronic consent via REDCap to participate in the full study. They also provided the information required for the study payment. Participants then completed physiological measurements (urine cotinine) and uploaded a photograph of their results to confirm smoking status. If a participant’s cotinine test result was not above the threshold required for eligibility, the participant was excluded from continuing in the study.

Participants who met the urine cotinine threshold for eligibility then completed self-report measures (see Table 1, with additional information available at GitHub [49]). They read online instructions for the baseline period EMA task and answered comprehension questions to ensure that they understood the task. Participants also installed the RealLife Exp app (the EMA app) on their smartphone. During EMA setup, participants chose 1 of 4 possible EMA start times (6 AM, 8 AM, 10 AM, or 12 PM). They then completed a practice EMA session. The session was administered over REDCap and Qualtrics, and was expected to take less than 1 hour.

**Table 1.** Study questionnaires by study timepoint^a^.

Category and questionnaire	Session 1	Session 2	Session 3	Scan
Smoking specific				
Smoking behavior questionnaire^b^^c^	✓	✓	✓	✓
Fagerström test of nicotine dependence	✓	✓	✓	✓
Smoking habits^b^^c^	✓		✓	
Cessation intentions^b^^c^	✓		✓	
Smoking beliefs^c^	✓		✓	
Smoking attitudes^c^	✓		✓	
Smoking norms^c^	✓		✓	
Social motivations for smoking	✓			
Smoking self-concept scale^c^	✓		✓	
Smoking information exposure^b^^c^	✓	✓		
Health and well-being				
Psychotropic drug use^b^	✓			
Alcohol use^c^				✓
Center for Epidemiologic Studies Depression (CES-D 10)	✓			
Stressful Life Experiences Inventory		✓		
Perceived Stress Scale	✓		✓	
Mindful Attention Awareness Scale				✓
Purpose in Life Scale^b^	✓			
Perceived microaggressions^c^	✓		✓	
MRI^d^ scan screening	✓			✓
Other				
Demographics^b^^c^	✓		✓	
MacArthur Scale of Subjective Social Status	✓		✓	
Code switching^b^	✓		✓	
BIS-11^e^				✓

a Questionnaires are available on GitHub [50].

b Questionnaire items or scales developed in the context of this study that have not been psychometrically validated.

c Adapted measures.

d MRI: magnetic resonance imaging.

e BIS-11: Barratt Impulsiveness Scale.

#### Step 2: Baseline Period

This period began 2 days after the completion of Online Session 1, and lasted for 14 days. During the baseline period, participants completed EMA and location tracking. Participants completed two kinds of EMA surveys: (1) “check-ins,” which were sent 4 times per day, and (2) “summaries,” which were sent 2 times per day. The summary surveys were sent at the same 2 times every day (midday and end of day), depending on the participant’s chosen start time. Two of the check-in surveys were sent at the same time as the summary surveys, and the other two were signal contingent, sent randomly within designated 4-hour time windows (one before the first summary survey, and one between the first and second summary surveys). Participants therefore received survey requests 4 times per day. If participants did not complete any EMA surveys during the first few days of the study, they were contacted to confirm they were receiving the surveys and that the RealLife Exp app was set up correctly.

Cigarette craving and smoking assessment items measured the primary outcomes of this study. The craving prompt item was, “Right now, how much are you craving a cigarette?,” with responses on a sliding scale in increments of one from “0=not at all” to “100=extremely.” The smoking prompt item was, “Between [prior time] and [current time], how many cigarettes have you smoked in total?” and participants used an open-ended numeric entry. Additional EMA items are described in Table 2 and in detail on the study website [49]. The study protocol combined signal contingent and coverage sampling approaches to balance the goals of capturing fluctuations in momentary cravings experienced throughout the day (which is best assessed by signal contingent sampling) and of accurately measuring smoking behavior (which is best assessed by coverage, or fixed interval, sampling). Signal-contingent sampling can reduce participants’ ability to predict when the next prompt will arrive and elicit more natural reporting [51]. Prior work has found that coverage reporting generates consistent and accurate reporting about cigarette smoking [52-55].

**Table 2.** Questionnaires administered via ecological momentary assessment, by study timepoint.

Category and questionnaire	Baseline daily	Intervention daily	Intervention weekly
Smoking-specific			
Craving^a^	✓	✓	✓
Smoking^a^	✓	✓	
Social smoking behavior^a^			✓
Smoking information exposure^a^^b^			✓
Conversations (smoking)^a^	✓	✓	
Well-being			
Conversations (general)^a^	✓	✓	
Positive and Negative Affect Schedule I-SF^c^	✓	✓	
Stress^a^^b^	✓	✓	
Purpose in Life Scale^a^	✓	✓	
Loneliness and social support^a^			✓
Perceived microaggressions^b^	✓	✓	

a Questionnaire items or scales developed in the context of this study that have not been psychometrically validated.

b Adapted measures.

c I-SF: International Short Form.

#### Step 3: Online Session 2

Participants were invited to complete Online Session 2 after completing the baseline period. They were encouraged to complete the session within 1 week of receiving the invitation. Participants completed self-report measures (see Table 1), an image rating task, geolocation data export and upload, EMA setup and practice for the intervention period, and instructions and practice for the intervention tasks. Online Session 2 was administered over Qualtrics and was expected to take less than 1 hour.

In the image rating task, participants viewed photographs and were asked to think about whether the images made them want to smoke a cigarette. Participants saw 35 sets of unique image pairs and rated their craving after each set on a scale from 0 (“not at all”) to 100 (“extremely”). Each image was shown for 3 seconds. The craving rating screen also included a checkbox for “unable to see the image(s),” so that participants could report any technical issues. There were 7 categories of images. Image pairs were within the same category, and the presentation order of the categories was randomized. Each category was repeated 5 times, and each image shown was unique. The categories of images were (1) standard smoking cues (eg, cigarettes in a pack, in an ashtray, or alongside a lighter), (2) compositionally similar nonsmoking cues (eg, pencils in a pack, with a sharpener, or sitting on a table), (3) views of the cash register area at several nontobacco retail stores, (4) views of the cash register and power wall area at several tobacco retail stores, (5) close-up photographs of a single brand of cigarette packs, (6) compositionally matched photographs of a single brand of cigarette packs with a price promotion feature, and (7) close-up photographs of packs of gum with a price promotion feature. Standard smoking and nonsmoking cues were customized to show either brown or white filters, chosen to match the filter color of the participant’s preferred cigarettes. Cigarette brands were also customized based on participant preferences (selected brand in response to “What brand do you prefer to smoke?,” with options L&M, Pall Mall, Camel, Newport, and Marlboro). Before beginning the image rating task, participants were asked how long ago they had smoked a cigarette and their current level of craving. They were also asked to refrain from smoking for the duration of the task (approximately 12 minutes).

#### Step 4: Intervention Period

The intervention period began after completion of Online Session 2. During this 4-week period, all participants completed EMA (see Table 2), location tracking, and weekly surveys. Participants were assigned to 1 of 3 experimental conditions: the tobacco retail condition, the nontobacco retail condition, or the control condition. Those in the tobacco retail and nontobacco retail store conditions were asked to enter an assigned retail store 5 times per week for 4 weeks. They were provided funds for spending US $3 per visit, which was loaded onto the participant’s Greenphire Clincard, and they were asked to make a small purchase (excluding tobacco products). Using their cell phone, participants were asked to submit a photograph of their receipt via the RealLife Exp app on their phone after each store visit. In the tobacco retail condition, participants were assigned to enter a tobacco-selling convenience store chain within the study area (PA, DE, or NJ), and in the nontobacco retail condition, participants were assigned to enter a nontobacco-selling pharmacy/retail store chain within the study area. Those in the control condition were not asked to visit any additional store but received equal compensation and otherwise completed the same tasks.

#### Step 5: Online Session 3

Following the intervention period, participants were invited to complete Online Session 3, which they were encouraged to complete within 1 week of receiving the invitation. During Online Session 3, participants completed surveys (see Table 1), the image rating task, and geolocation data export and upload. At the end of the session, participants were instructed on how to uninstall study-related smartphone apps and turn off location tracking, as desired. Participants were also provided with a debrieﬁng form, which included details about the study procedure and research goals. They were also provided with information about services that provide smoking cessation assistance. For participants interested in being screened for the fMRI scan, debriefing and provision of the cessation resources document occurred after the scan or as soon as scan session ineligibility was determined; however, in most cases, participants were debriefed before the scan session. Participants who did not reply to the Online Session 3 invitation after several contact attempts were sent their final payment and information about smoking cessation and study debriefing.

#### Step 6: fMRI Session

Participants who completed Online Session 3 and were potentially eligible to participate in the optional, in-person fMRI session (as determined by self-report questions in previous screening surveys and online sessions) were invited to complete an fMRI screening survey. The fMRI scan procedure was added to the protocol in October 2022. If eligible based on the criteria described above, participants were scheduled for a 2-hour, in-person session at the University of Pennsylvania. Experimenters confirmed that participants met any COVID-19 screening requirements per the university and hospital system guidelines (eg, symptom screen, temperature check), then reviewed the consent addendum document. After providing electronic informed consent, participants provided a urine sample to confirm eligibility and completed any required forms (eg, W-2, additional metal screen) and surveys (Table 1). Eligible participants received safety and task-related instructions and training before completing the fMRI scan. During the 1-hour scan, participants completed an image rating task, similar to the task they completed during Online Sessions 2 and 3. In the fMRI version of the image rating task, participants saw 70 blocks of 4 images. Each image was shown for 4 seconds, and participants had 3 seconds to rate their craving on a scale from 1 (not at all) to 5 (very much) following each block. Participants viewed the same 7 categories of images as in the online task, and each category was repeated 10 times; different from the online task, each image was repeated twice during the fMRI session. In addition to the image rating task, participants completed anatomical scans (T1-weighted and T2-weighted) and a fieldmap scan for correction of inhomogeneities in the magnetic field. Scanning parameters can be found in Multimedia Appendix 1.

### Intervention Implementation

#### Randomization

Participants who completed the required tasks during the baseline period and attested to being able to complete store visits before beginning the intervention were randomized to an intervention condition. Participants were assigned to be in one of three conditions that manipulated their exposure to the retail environment: tobacco retail store, nontobacco retail store, or control (no store). The assignment was generated through blocked randomization, where the blocks were defined by gender (male, female, and other gender identities) and smoking level, which was dichotomized as high (20 or more cigarettes per day) or low (less than 20 cigarettes per day) as reported at Online Session 1. At the beginning of data collection, condition assignment was fully random; blocked randomization was implemented partway through the study on December 1, 2022. This resulted in a total of 66 participants being randomized prior to blocked randomization and 216 participants being randomized under blocked randomization.

Assignment to condition was implemented by the lead study research coordinator, using Microsoft Excel’s random number generator. Condition assignment did not occur until participants completed the baseline period, and the study team confirmed that the participant met all eligibility criteria for continuing into the intervention period (EMA response rate, upload of geolocation data from the baseline period). Given that multiple participants were always enrolled simultaneously, and it was not clear which participants would continue to be eligible following the baseline period, it was not possible for the study team to know with certainty which condition each participant would be assigned to until assignment had irrevocably occurred. Once the condition was irrevocably assigned, the assignment was stored in REDCap under each participant’s respective record, which was only accessible to the study team.

#### Blinding

The study team was aware of participants’ assignment to condition once it was assigned, allowing team members to provide appropriate instructions and assistance to participants specific to condition assignment. Participants were provided instructions based on condition but were not informed about the experimental purpose or condition assignment. For example, participants assigned to the tobacco retail condition were instructed to enter a specific tobacco-selling chain convenience store but were unaware of the study design and hypotheses. Participants were not informed of condition assignment, experimental purpose, and other study information until they received the debriefing handout after study completion.

#### Strategies to Improve Adherence

The protocol featured a number of strategies to encourage participant retention and study adherence. To ensure that study procedures were clear, the study team provided study appointment calendars or outlines to individual participants, reminder SMS text messages the day prior to and the day of scheduled calls and online sessions, reminder push notifications for EMA surveys, and regular text updates about study progress throughout the duration of the study. The online surveys included practice tasks, such as for EMA start-up and receipt submission, as well as brief quizzes to ensure that participants understood the instructions. Study instructions featured infographics to promote engagement with and understanding of the study materials [56].

To remove barriers for technical issues, the study team offered to assist with any technical difficulties or points of confusion when participants were observed to be unresponsive to study messages. There was a link to a form requesting live research support on every online survey page. To incentivize adherence to the protocol, participants were provided with incentives for completing each online session, larger payments at the final session, and a bonus for completing the entire study (excluding the optional fMRI scan). If participants decided to withdraw from the study, they were offered compensation to upload their geolocation data without being required to complete other study surveys for the relevant study time point. Individuals were able to pause their participation in the study in between sections to accommodate unforeseen travel.

#### Feasibility and Acceptability Assessment

Feasibility and acceptability of the study were assessed through extensive piloting, prior to issuance of the grant (1R01CA229305) that funded the project, and iteratively as the study team adapted to obstacles such as the onset of the COVID-19 pandemic. As described in Multimedia Appendix 1, the original goal of the project was for all participants to undergo fMRI scans; this was no longer feasible after the COVID-19 pandemic began, and the study team designed the remote version of the protocol described here. Retention rates in the study were high, speaking to the feasibility and acceptability of the protocol: 93.7% (282/301) from Online Survey 1 to Online Survey 2 (baseline period), and 86.5% (244/282) from Online Survey 2 to Online Survey 3 (intervention period), despite difficulties with adherence to the intervention (described in Results).

### Outcome Measures

#### Primary Outcome Measures

The study team preregistered primary outcomes and analysis plans for the intervention and fMRI scan portions of the study on ClinicalTrials.gov (NCT04279483) as well as on the Open Science Framework (OSF) [57,58], and also preregistered analysis plans for the correlational, baseline period of the study on OSF [59]. The primary outcomes for the baseline period of the study (core aim 1) were the associations between tobacco retailer exposure with cigarette craving and cigarettes smoked. The primary outcomes for the intervention period (core aim 2) were changes in cigarette craving and smoking as a function of the interaction between study phase (baseline vs intervention) and condition assignment (tobacco retail, nontobacco retail, control). In the fMRI subsample, the primary outcome was brain activity in response to smoking versus nonsmoking cues (core aim 3).

#### Exploratory Covariates and Outcome Measures

The team collected additional survey data to address exploratory analysis questions. This included personality measures (collected at the online sessions) and affect ratings (collected during the daily EMA surveys), among many other survey items; study instruments are summarized in Tables1  2, and can be found at GitHub [49]. These data provide opportunities to investigate how other psychological variables may be associated with smoking behavior and how these variables may act as mediators or moderators of relationships between exposure to tobacco retail and smoking behavior.

### Statistical Analysis Plans

For the first core aim, within-person associations between daily tobacco retail exposure, daily cigarette craving, and daily smoking during the baseline period were analyzed using multilevel models, in line with the publicly posted preregistration [59,60]. Daily craving was calculated by computing the mean value of all craving ratings (4× per day) each day. The total of daily cigarettes smoked was calculated by summing self-reported cigarettes (reported 2× daily). For missing assessments, the cigarettes smoked variable was imputed using either the participant’s average during the first or second assessment, aligning with the prompt missed. Tobacco retail exposure was calculated by joining participants’ geolocation data with the locations of tobacco retailer licenses, using a multistep algorithm to compute exposures [60], and summing the exposures by day. To investigate within-person and between-person associations, tobacco retail exposure was split into both a time-invariant, between-person variable and a time-variant, within-person component. To account for time, day-in-study was included as a covariate. Sensitivity analyses were performed to investigate whether associations were influenced by the covariates of race, ethnicity, gender, age, nicotine dependence, smartphone type, and state of residence.

For the second core aim, the causal effects of tobacco retail exposure on cigarette craving and smoking will be analyzed using data collected during both the baseline and intervention periods [58]. For each participant, the mean value of daily craving ratings and the mean value of cigarettes smoked per day will be calculated separately for the baseline and intervention periods. A study period variable will indicate whether the observation occurred during the baseline versus intervention period, and two dummy-coded variables will indicate the condition assignment for each participant. Using a multilevel model, the interaction between the study period and condition assignment will be tested. An intent-to-treat approach will be the primary model, including all participants regardless of intervention compliance. In an alternative model, analysis will only include participants with 100% intervention compliance. Additional alternative models will assess if effects vary by compliance level, by including the number of intervention days completed as a covariate, and by only including participants with a predetermined number of intervention days completed. Sensitivity analyses will be performed to investigate whether findings are influenced by covariates such as race, ethnicity, gender, age, nicotine dependence, smartphone type, state of residence, and exposure to other point-of-sale tobacco marketing. Further, additional sensitivity analyses will be conducted to account for protocol changes that occurred during data collection. For instance, including variables that indicate whether participants were enrolled prior to, or after protocol changes, will permit investigation of whether results are robust when accounting for protocol modifications including the introduction of the intent-to-treat approach instead of excluding for noncompliance during the intervention, adding a prerandomization screen about the ability to complete store visits, and switching to blocked randomization.

For the third core aim, behavioral and neural data collected during the image rating fMRI task at the optional fMRI session will be analyzed to investigate whether cue reactivity is an explanatory mechanism for associations between naturalistic marketing exposure and craving and smoking. Multilevel models will test whether self-reported craving will be greater in response to viewing tobacco retail images compared to standardized nonsmoking images and compared to nontobacco retail images [57]. Similarly, multilevel models will test whether averaged neural activity in smoking cue reactivity regions will be greater in response to viewing tobacco retail images compared to standardized nonsmoking images and compared to nontobacco retail images. Sensitivity analyses will be performed to investigate whether findings are influenced by covariates such as ethnicity, gender, age, nicotine dependence, smartphone type, state of residence, and intervention period condition assignment.

### Sample Size

The initial target was to obtain 60 complete datasets per intervention condition (meaning that participants finished Online Session 3 and also completed all 20 store visits; total n=180). Due to pandemic-related resource constraints and time delays, this goal was impossible. The revised study design collected data from the maximum number of participants possible with the resources available (in the tobacco retail condition, n=56 with all 20 store visits; in the nontobacco retail condition, n=44 with all 20 store visits). More individuals were randomized to the experimental conditions than to the control condition, since less than 50% of participants typically completed all 20 store visits and provided complete datasets. Before conducting any hypothesis testing, the biostatistician reassessed power in consultation with the study team to determine which original hypotheses would be adequately powered with the available data and planned analysis methods. Using PANGEA [61], it was estimated that with at least 40 complete datasets (participants who completed all 20 store visits) in each intervention condition, effects of size Cohen *d*=0.1 would be detectable with 95% power. Ultimately, 105 participants were randomized to the nontobacco retail condition, 107 participants to the tobacco retail condition, and 70 participants to the control condition.

### Ethical Considerations

#### Ethical Approval

The study protocol (protocol number 850796) was approved by the University of Pennsylvania’s IRB on February 3, 2022. The study was also approved by the Clinical Trials Scientific Review (ClinicalTrials.gov Identifier: NCT04279483) and Monitoring Committee at the University of Pennsylvania’s Abramson Cancer Center (UPCC 08022). All participants provided informed consent and were paid for their participation.

#### Consent

Informed consent was collected at multiple stages throughout the study. Participants signed an electronic consent form at the start of Online Screen B, which followed the initial phone screening. Participants who were potentially eligible following Online Screen B were directed to Online Survey Session 1, in which participants provided their informed consent electronically to participate in the full study. Participants who completed the baseline and intervention components and who were interested in the fMRI component provided electronic consent at the beginning of the fMRI screening survey. Eligible participants who completed the fMRI scan provided consent at the beginning of the session.

#### Confidentiality

All data collected through this study were treated as strictly confidential. Data were deidentified as much as possible and were accessible only to select study team members. Deidentified data were coded using a dual ID system: Online Survey A automatically assigned a REDCap record ID number to each screen respondent who expressed interest in participating. This number was different from the study ID number, which was only assigned to enrolled participants (those who signed the full electronic informed consent on REDCap administered during Online Session 1). Participants were informed that this research was covered by a Certificate of Confidentiality from the National Institutes of Health. This means that the researchers cannot release or use information, documents, or samples that may identify the participant in any action or lawsuit unless the participant approves. This protection includes federal, state, or local civil, criminal, administrative, legislative, or other proceedings.

#### Data Monitoring

No data monitoring committee was needed as the principal investigator and study team continuously monitored the privacy and integrity of the data throughout the study. In accordance with the IRB protocol, the study team monitored for serious adverse events in real time, and recorded and reported any serious or other adverse events. Following the conclusion of their participation in the study, every participant was provided with information about local services that provide smoking cessation assistance, smoking studies at Penn, quitline resources, online quit smoking resources, and specific quitting tips.

#### Stopping Rule

A stopping rule was implemented such that at 2 points during the study, the principal investigator and team assessed whether the intervention was causing harm to participants. After data collection from 60 participants and 120 participants, the study team assessed the number of individuals in the tobacco retail condition who had an extreme increase in smoking compared to individuals in the control condition. If an individual’s average daily cigarette consumption during the final 2 weeks of the intervention phase was greater than 3 SDs above their average daily cigarette consumption during the baseline phase, they were considered to have an extreme increase in their smoking. If the number of individuals in the tobacco retail condition who had an extreme increase in smoking was significantly (α=.01) greater than the number of individuals in the control condition who had an extreme increase in smoking, the study team planned to reconvene to consider termination of future assignment to the tobacco retail condition. However, this criterion was not met at either time point, and hence, no participants were impacted by this rule.

#### Harms

All harms and adverse events were reported to the University of Pennsylvania’s IRB and to study sponsors in accordance with standard policies of these respective institutions.

### Data Collection and Management

#### Data Collection Sources

Data were collected via different platforms: EMA data were collected via LifeData, self-report questionnaire data were collected via REDCap and Qualtrics, geolocation data were collected through Google Maps, and participants subsequently downloaded and uploaded their data files via Qualtrics. Image rating fMRI task data were collected via a computer-programmed Python task, and brain data were collected using a 3-tesla fMRI scanner. Study instruments can be found at GitHub [49].

#### Data Management

Identifiable information and survey data were stored in the project’s REDCap database. EMA data collected via LifeData were unidentifiable, and each participant was assigned a user ID that was different from the study ID, but whose match was stored in the REDCap database. All data from LifeData were stored on a password-protected server that was only accessible to select study team members. Geolocation data, data from the image rating task, and fMRI data were also stored on the same password-protected server. Code created for the preprocessing and analysis of data is maintained on a private GitHub repository to enable version-control tracking and collaboration within the study team.

#### Tobacco Retail Database

The study team curated a database of all tobacco retail stores across PA, NJ, and DE. Tobacco retail information was publicly available through open data sites for PA [62] and DE [63], and updated retail lists were downloaded monthly by the study team. Tobacco retail information for NJ was only available upon request, which the study team requested annually. Tobacco retail lists from each state consisted of store name, license number, license type, and street address. The study team constructed a custom codebase to preprocess the retail data. For instance, one feature adds the latitude and longitude coordinates of the NJ retail stores (which are not provided by the state) or of other retail stores missing coordinates based on the provided street addresses, using the Google Places API. This enables further cross-referencing between retail locations and participant locations collected via Google Maps. License start and expiration dates were also generated based on retail stores appearing or being removed in newly published databases. The retail database can be found on GitHub [49]. In total, the custom database contained 36,580 retail stores, including 23,293 in PA, 11,843 in NJ, and 1444 in DE. In cases where the retail location was incorrectly provided, but recovering the correct location was feasible, the study team manually obtained the corrected geolocation via Google Maps and updated the database. Examples of cases that prompted further investigation include a missing address, an address outside of the 3 states, a nonexistent address, and many retail stores with the same address.

### Dissemination and Data Availability

#### Dissemination Plans

Findings from this study are being disseminated in the following ways: preprint articles, peer-reviewed journal articles, conference presentations, seminars and colloquia, public talks and interviews, press releases, and social media posts. The study team also plans to discuss the findings with a community advisory board as further dissemination plans are refined, to make the work most useful to the communities that participated in the work, and to policymakers.

#### Data Availability

Final research data, with all identity-related information deleted and in consultation with the relevant IRBs, will be made available to the scientific community for collaborative research with members of the study team, upon request. The data will be shared in spreadsheet format for all nonimaging data and in NIfTI (Neuroimaging Informatics Technology Initiative) format for fMRI data. Data will be shared upon request in consultation with relevant IRBs for further analysis, once the study’s primary results have been published. Qualified investigators who wish to access the study materials can complete a form on the project website [64], which describes the study and delineates the specifics of data use. The requests will be reviewed and approved by the investigators and by the relevant IRBs. To protect participant privacy, data that may be difficult to deidentify (including geolocation data) will be shared at an aggregate level, with restrictions as developed in consultation with the IRB at the University of Pennsylvania. The requested research data files will be accompanied by a description of variables and how they were collected. The study will also share protocols relevant to data collection procedures as needed, and in consultation with other interested researchers.

## Results

**Figure 2.**
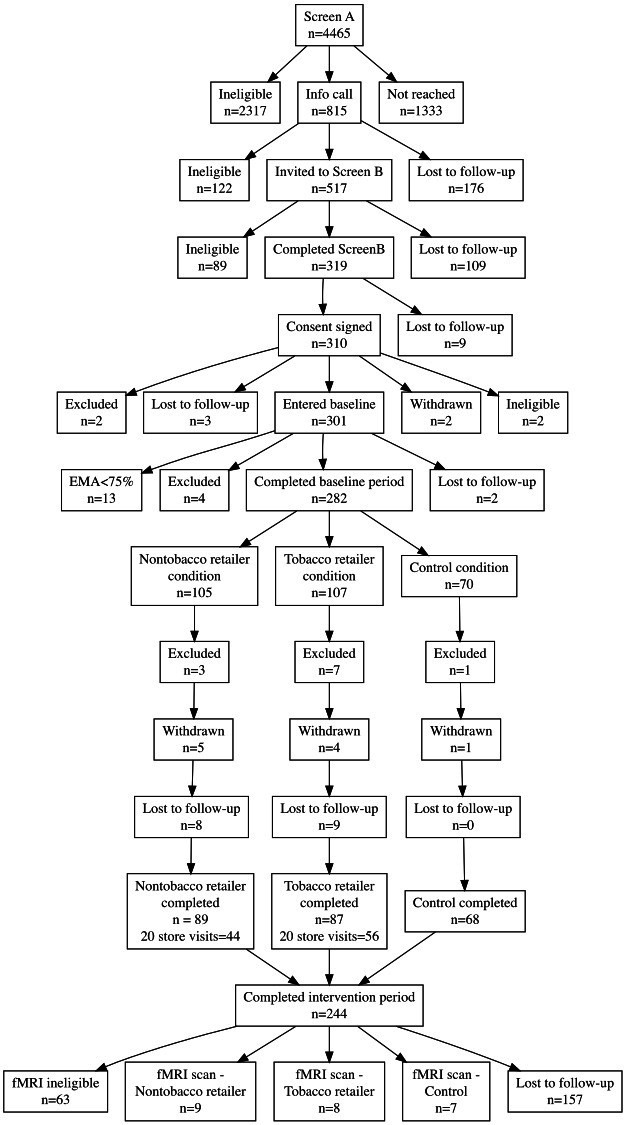
Participant retention flowchart. EMA: ecological momentary assessment; fMRI: functional magnetic resonance imaging.

The research protocol was approved by the IRB at the University of Pennsylvania on February 3, 2022. Data collection began on May 25, 2022, and was completed on June 10, 2024. A total of 310 adults who currently smoke cigarettes were enrolled. Of these, 282 participants completed the baseline phase, 244 participants completed the study, and 24 participants completed the optional fMRI session in the main protocol (see Multimedia Appendix 1 for details on the pilot study that includes additional data, including fMRI scans). See Figure 2 for a detailed overview of participant retention.

A total of 310 participants consented to participate and began the main study. After signing the consent and before beginning the baseline period, a total of 9 participants were determined ineligible for the following reasons: age (n=1), lack of physiological confirmation of smoking status (n=1), experienced technical difficulties (n=2), stopped responding (lost to follow-up) (n=3), and withdrew participation (n=2). After completing Online Session 1 and entering the baseline period, 19 participants were excluded due to low EMA response rates (n=13), inability to upload location history (n=4), and lost to follow-up (n=2). Following randomization, 11 participants were excluded during the intervention period: 5 were unable to participate in the store visits before this exclusion criterion was removed (2 assigned to the tobacco retail condition and 3 assigned to the nontobacco retail condition), 1 due to low EMA response rate in the tobacco retail condition before this exclusion criterion was removed, 4 due to technical issues (3 in the tobacco retail condition and 1 in the control condition), and 1 due to working at a tobacco retail (tobacco retail condition). A total of 10 participants withdrew from the study during the intervention period (4 in the tobacco retail condition, 5 in the nontobacco retail condition, and 1 in the control condition), and 17 participants were lost to follow-up (9 in the tobacco retail condition, 8 in the nontobacco retail condition, and none in the control condition). In total, 244 participants completed the intervention period and were invited to complete the optional fMRI study. Of these, 63 participants were ineligible and 157 were not interested. Therefore, 24 participants completed the fMRI (8 from the tobacco retail condition, 9 from the nontobacco retail condition, and 7 from the control condition).

**Figure 3.**
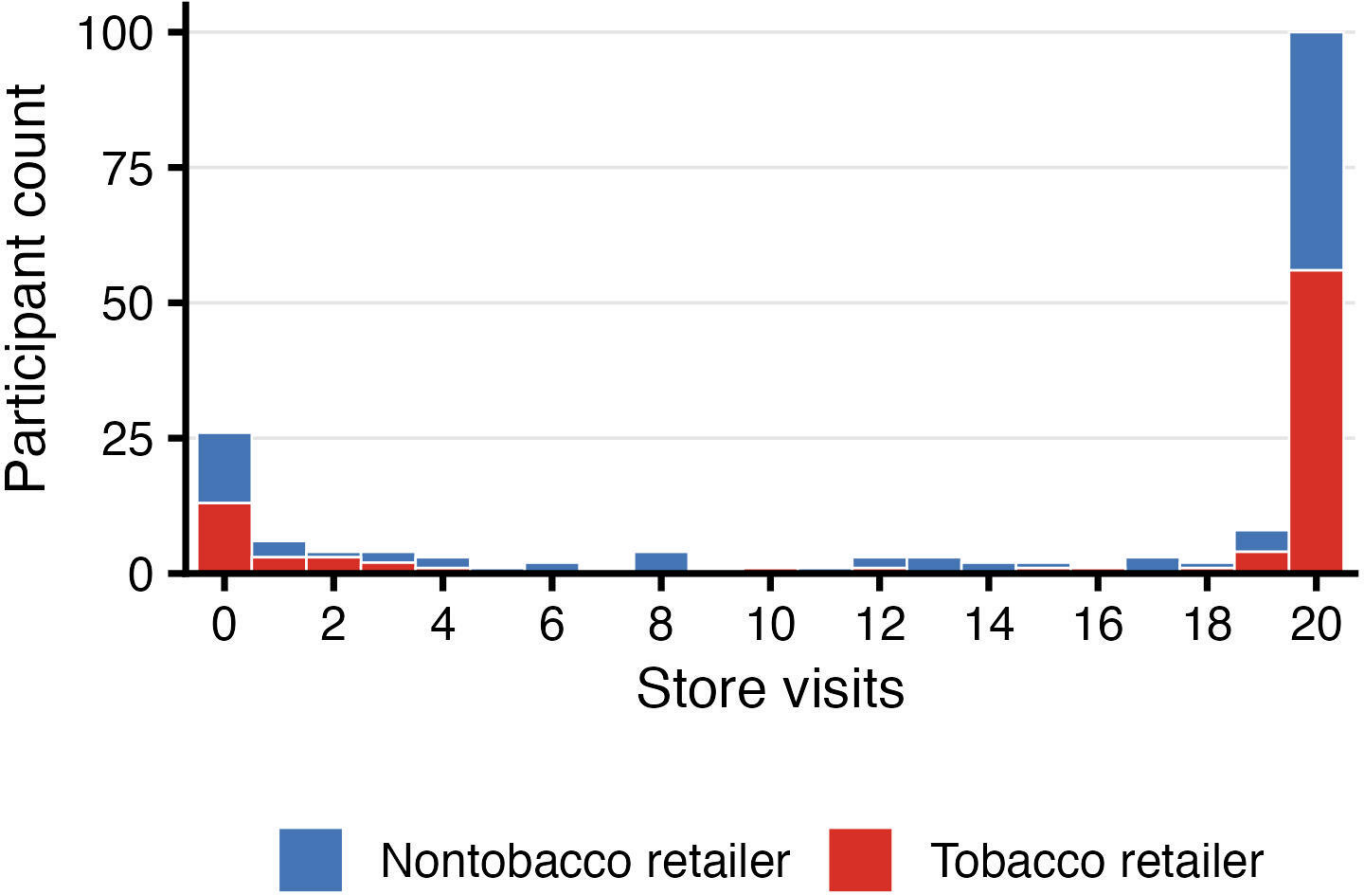
Distribution of intervention compliance.

Retention rates in the study were high: 93.7% (282/301) from Online Survey 1 to Online Survey 2 (baseline period), and 86.5% (244/282) from Online Survey 2 to Online Survey 3 (intervention period). Not all participants who completed Online Session 3 and the intervention period fully completed the intervention task (ie, completing all 20 store visits for participants in the store conditions). See Figure 3 for a histogram displaying the distribution of intervention compliance. Overall, in the tobacco retail condition, participants made an average of 14.8 (SD 8.3) store visits, and in the nontobacco retail condition, participants made an average of 13.6 (SD 8.1) store visits. In the tobacco retail condition, 56 (64%) participants made all 20 store visits, 13 (15%) participants made 0 visits, and 18 (21%) participants made between 0 and 20 visits (average=9.2). In the nontobacco retail condition, 44 (49%) participants made all 20 store visits, 13 (15%) participants made 0 visits, and 32 (36%) participants made between 0 and 20 visits (average=10.3). A total of 10 participants provided no data (EMA, geolocation, or store visit receipts) following completion of Online Session 3 (n=3 in the nontobacco retail condition and n=7 in the tobacco retail condition), and those participants were not counted in these averages. As noted above, when the study data collection began, participants who could not make all 20 purchases were excluded. However, partway through data collection (at 20% complete), the study protocol shifted to an intent-to-treat framework and did not exclude participants for noncompliance once they had been randomized to an intervention condition.

The rate of enrollment in the fMRI scan session was low. Although the original goal was to complete scans on all participants, the study protocol was adapted to a remote protocol when the COVID-19 pandemic began. Given that recruitment efforts did not target fMRI eligibility or proximity to the University of Pennsylvania, it is unsurprising that few participants were interested in and eligible for the fMRI scan.

Participant demographics at each stage of the study are described in tables. Table 3 presents demographics in the baseline period, separated by those who began the baseline period, those who were excluded for low EMA compliance during the baseline period, and those who completed the baseline period (by completing Online Session 2). Tables S1-S3 in Multimedia Appendix 1 present participant demographics in the tobacco retail, nontobacco retail, and control conditions (respectively), separated by those who completed Online Session 2 and began the intervention period, completed the intervention period and Online Session 3, and, for the tobacco retail and nontobacco retail conditions, those who completed all 20 store visits.

EMA response rates were high. Of the 301 participants who began the baseline period, only 13 participants (4.3%) had EMA response rates lower than 75% during the baseline period. Table 4 describes average EMA completion rates by study stage and condition.

Table 5 describes average daily geolocation points by study stage and condition. Geolocation data were missing or unusable for 9 participants who completed the baseline phase and 10 who completed the intervention period.

**Table 3.** Demographics for participants in the baseline period, separated by those who began the baseline period, those who were excluded because of low EMA compliance during baseline, and those who completed the baseline period (by completing Online Session 2). Demographics for those who completed the baseline period are also presented separately by those assigned to the tobacco retail condition, nontobacco retail condition, and control condition.

Variable	Began baseline period (n=301)	Completed baseline period (n=282)	Excluded during baseline (n=13)	Tobacco retailer condition (n=107)	Nontobacco retailer condition (n=105)	Control condition (n=70)
Age (in years), mean (SD)	42.7 (10.6)	42.9 (10.6)	40.7 (11.2)	44.2 (10.6)	42.5 (10.7)	41.4 (10.4)
Gender, n (%)						
Men	122 (40.5)	114 (40.4)	6 (46.2)	45 (42.1)	41 (39)	28 (40)
Women	168 (55.8)	157 (55.7)	7 (53.8)	58 (54.2)	59 (56.2)	40 (57.1)
Other	11 (3.7)	11 (3.9)	0 (0)	4 (3.7)	5 (4.8)	2 (2.9)
Race, n (%)						
American Indian/Alaska Native	2 (0.7)	2 (0.7)	0 (0)	1 (1)	0 (0)	1 (1.4)
Asian	7 (2.3)	6 (2.2)	0 (0)	0 (0)	3 (2.9)	3 (4.3)
Black	75 (25.2)	65 (23.3)	9 (69.2)	27 (25.7)	23 (21.9)	15 (21.7)
More than one race	14 (4.7)	14 (5)	0 (0)	2 (1.9)	8 (7.6)	4 (5.8)
White	189 (63.4)	183 (65.6)	3 (23.1)	73 (69.5)	66 (62.9)	44 (63.8)
Self-describe	10 (3.4)	8 (2.9)	1 (7.7)	2 (1.9)	5 (4.8)	1 (1.4)
Prefer not to say or not reported	4 (1.3)	4 (1.4)	0 (0)	2 (1.9)	0 (0)	2 (1.9)
MacArthur Scale of Subjective Social Status, mean (SD)	4.8 (1.9)	4.7 (1.8)	4.2 (2.8)	4.7 (1.9)	4.7 (1.7)	4.9 (1.7)
Cigarettes per day, mean (SD)	16 (10.4)	16.1 (10.6)	15.9 (7.3)	16.9 (14.4)	15.5 (7.6)	15.7 (7.1)

**Table 4.** EMA^a^ response rates.

	Completed baseline period			Completed intervention period			Completed intervention period with 20 store visits		
	Responses, n	Participants, n	EMA response rates, mean% (SD%)	Responses, n	Participants, n	EMA response rates, mean% (SD%)	Responses, n	Participants, n	EMA response rates, mean% (SD%)
*Full group*	*13,956*	*282*	*92.8 (6.8)*	*23,385*	*244*	*89.7 (12.6)*	*99,811*	*100*	*94.9 (5.8)*
Tobacco retailer	5254	107	93.0 (6.9)	8448	87	89.7 (14.3)	5833	56	94.7 (6.3)
Nontobacco retailer	5210	105	93.2 (6.5)	8267	89	88.8 (12.8)	4148	44	95.2 (5.1)
Control	3492	70	91.9 (7.1)	6670	68	90.7 (9.7)	—^b^	—	—

a EMA: ecological momentary assessment.

b Not applicable.

**Table 5.** Daily average geolocation point observations.

	Completed baseline period			Completed intervention period			Completed intervention period with 20 store visits		
	Observations, n	Participants, n	Mean (SD)	Observations, n	Participants, n	Mean (SD)	Observations, n	Participants, n	Mean (SD)
*Full group*	*1,181,820*	*273*	*311.4 (296.2)*	*1,992,886*	*234*	*306.8 (292.1)*	*763,881*	*93*	*295.2 (256.6)*
Tobacco retailer	472,951	104	328.5 (337.5)	746,019	85	314.9 (300.6)	426,481	54	283.4 (275.8)
Nontobacco retailer	412,570	101	293.4 (254.9)	693,162	84	298.9 (251.9)	337,400	39	311.5 (229.7)
Control	296,299	68	311.8 (288.3)	553,705	65	306.5 (330.9)	—^a^	—	—

a Not applicable.

## Discussion

### Anticipated Findings

The multimodal GeoSmoking study protocol was designed to understand how exposure to people’s real-world environments relates to their experiences of craving and smoking, and the brain mechanisms that link exposure to key outcomes. This study protocol has been implemented, and data collection is complete.

The first core aim of the study was to examine within-person associations between real-world tobacco retailer exposure and cigarette craving and smoking. One hypothesis was that individuals would report higher cigarette cravings on days when their exposure to tobacco retailers was higher than usual. Another hypothesis was that individuals would report smoking more cigarettes on days when their exposure to tobacco retailers was higher than usual. Both hypotheses were supported, as described in Muzekari et al [60]. These results are in line with previous work describing the detrimental effects of exposure to tobacco retailers on individuals who smoke. The GeoSmoking study expanded on that prior work by examining these effects dynamically within individuals, over an extended period, within individuals’ natural daily environments. This provides additional evidence for the detrimental effects of tobacco retail marketing on smoking outcomes.

The second core aim of the GeoSmoking study was to examine the causal effects of real-world tobacco retailer exposure on cigarette craving and smoking. The primary hypothesis was that reported cigarette craving and cigarettes smoked would be higher for individuals in the tobacco retailer condition, relative to the nontobacco retailer and control conditions, during the intervention phase but not the baseline phase (see hypothesis preregistration [61]). Analysis of this hypothesis is ongoing. Policy efforts targeting the limitation of tobacco retail marketing, such as marketing bans and regulated or hidden displays, might be effective measures to guard against detrimental effects on individuals who smoke, but more research is needed that offers both longitudinal, ecologically valid designs and evidence of causal relationships between repeated exposure to point-of-sale tobacco marketing and smoking [65]. If increased exposure to tobacco retail is found to causally increase craving and/or smoking, this would provide greater support to policy change efforts.

The third core aim of the study was to examine neural cue reactivity as an explanatory mechanism for how real-world marketing affects craving and smoking. To address the question of whether tobacco marketing serves as a smoking cue, analyses will compare craving ratings and activity in regions of the brain that are responsive to smoking cues in response to three pairs of conditions from the image rating task. The first hypothesis is that craving ratings and neural cue reactivity will increase in response to images of standardized cigarette cues relative to images of standardized noncigarette cues, confirming that the standardized images are eliciting the expected responses. Next, the second hypothesis is that craving ratings and neural cue reactivity will increase in response to images of tobacco retail (photos of the cash register area at a convenience store with tobacco marketing and products) relative to standardized noncigarette cues (a control condition). Finally, the third hypothesis is that craving ratings and neural cue reactivity will increase in response to images of tobacco retail relative to nontobacco retail (photos of the cash register area at a pharmacy store without tobacco marketing and products), testing whether tobacco retail elicits stronger cigarette craving responses than other types of retail. For further details about neuroimaging analysis plans, see the preregistration [57]. Insight into the neural processes underlying cue-reactivity to tobacco retail will help to build mechanistic models explaining real-world effects, and can inform behavior change and neurofeedback interventions.

Further, in studies combining neuroimaging and smartphone data, neural data can be used in brain-as-moderator approaches [66]. For instance, the GeoSmoking study data can be used to answer questions such as whether in-scanner brain activation is related to individuals’ real-world levels of tobacco retail exposure, or whether relationships between tobacco retail exposure and smoking outcomes are stronger for individuals with greater neural cue-reactivity. Thus, the neural data not only provide mechanistic insight but can increase the explanatory and predictive power of naturalistic smoking behavior.

### Study Strengths and Limitations

Multiple strengths position this study to have a meaningful impact on tobacco control research. This work used real-time measurement of momentary experiences and geolocation repeated over time within a given individual, in that individual’s everyday environments. This approach is rare in prior work and allows examination of previously unanswered questions about day-to-day variability in individuals’ experiences, exposure to retail tobacco marketing, and smoking. Beyond the investigations of correlational associations during the baseline period, the study protocol manipulated tobacco retail exposure during a 4-week intervention, which allows for testing for potential causal effects of exposure to tobacco retail on craving and smoking. This real-world experimental approach both enhances internal and ecological validity and builds on previous experimental studies that used less naturalistic designs.

In addition to its strengths, the GeoSmoking study also has limitations. Generalizability of the study population is limited by the overrepresentation of individuals identifying as non-Hispanic White (189/301, 62.8%) and women (168/301, 55.8%), relative to the US average for adults who smoke cigarettes [67]. Although the majority of participants completed a significant portion of the store entry intervention, only 56.8% (100/176) of participants were fully compliant, completing all 20 store visits. The primary planned analyses for assessing the intervention will use an intent-to-treat approach, and secondary analyses will account for the variability in compliance with the intervention [58]. Low accrual into the fMRI scan arm of the study also limits statistical power for examining neural hypotheses.

Using state-of-the-art methods such as geolocation tracking poses challenges, including a lack of established standards for analysis. There are currently no clear best practices for the definition of exposure to risk factors (like tobacco retail) in people’s daily environments, with temporal and spatial boundaries having varied definitions across published work [68-71]. Thus, the current protocol and proposed analyses can inform such standards. The approach is limited in its capacity to detect whether exposure results from participants being inside the tobacco retail store versus passing by the store exterior. Thus, the protocol may overestimate exposure to retail tobacco marketing because not all stores feature outdoor tobacco advertising. Considering the stimuli used in the fMRI paradigm are images of tobacco marketing inside the point-of-sale, estimates of reactivity in the lab versus the real world are not a perfect match. Future research could assess whether interior tobacco advertising is visible from outside of each tobacco retail store, but this is resource-intensive to conduct at a large scale.

### Future Directions

The GeoSmoking study focused recruitment in PA, NJ, and DE due to the availability of tobacco retailer information in those states and the potential for participants to travel to Philadelphia for fMRI scans. Future work could replicate the GeoSmoking study in other locations to test whether the effects of exposure to tobacco retailers vary by geographic region within the United States. The density of tobacco retailers is particularly high in the Philadelphia area [72,73], and relationships between exposure to tobacco retailers and smoking outcomes may differ in locations with less dense retail environments.

Future work could adapt findings from the GeoSmoking study into actionable recommendations for smoking reduction or cessation interventions, including just-in-time interventions. This style of intervention is delivered, typically via mobile phone, at times when individuals might be most vulnerable to increased smoking or to relapse during a quit attempt [74,75]. If integrated with maps of tobacco retailer locations, tailored support could be sent via an individual’s smartphone when they are in proximity to tobacco retailers.

This approach to developing a comprehensive framework for understanding individuals’ daily experiences can also be applied to a broad range of factors in the environment, beyond exposure to tobacco retail. For example, future work might investigate exposure to other forms of retail advertising (such as alcohol) or to a multitude of factors in the built, natural, or social environment. Future research could also incorporate more qualitative methodology, including participant interviews, to further understand individuals’ experiences of their daily environments and how exposure to those environments influences their health-related decision-making.

### Conclusions

The GeoSmoking study was multidisciplinary, incorporating methods and perspectives from psychology, communication, geographic information systems, and neuroscience to assess the effects of real-world tobacco retail exposure on cigarette craving and smoking. Using both correlational and experimental approaches, the project assessed how exposure to tobacco retail in people’s daily lives affects cigarette smoking, the world’s leading cause of tobacco-related disease and death. Findings have the potential to inform cancer prevention efforts through future research, smoking cessation interventions, and policy changes.

## Supplementary material

10.2196/89627 Multimedia Appendix 1 Supplementary protocol and demographic information.

## Abbreviations

DE: Delaware
EMA: ecological momentary assessment
fMRI: functional magnetic resonance imaging
IRB: institutional review board
NIFTI: Neuroimaging Informatics Technology Initiative
NJ: New Jersey
OSF: Open Science Framework
PA: Pennsylvania
REDCap: Research Electronic Data Capture
