# Supplementary material for: Using Ecological Momentary Assessment, Geolocation Tracking, and Neuroimaging to Assess Effects of Tobacco Retail Exposure on Smoking Behavior: Protocol for the GeoSmoking Study
Source: JMIR Res Protoc. 2026 Jul 10;15:e89627. doi: 10.2196/89627 (PMC13353911; doi:10.2196/89627)

# Supplementary protocol and demographics information:

**fMRI Scanning Parameters**

Functional images and a fieldmap scan were collected using the following parameters: TR = 3,000 ms; TE = 32 ms; flip angle = 90 degrees; 46 axial slices; FoV = 252 mm; slice thickness = 3 mm; voxel size = 3 x 3 x 3 mm. T1-weighted images were collected using the following parameters: MPRAGE; TR = 1,850 ms; TE = 3.91 ms; flip angle = 8 degrees; 160 slices; FoV = 240 mm; slice thickness = 1 mm; voxel size = 0.9 x 0.9 x 1.0 mm. T2-weighted images were collected using the following parameters: TR = 3,200 ms; TE = 408 ms; 176 slices; FoV = 250 mm; slice thickness = 1 mm; voxel size = 1 x 1 x 1 mm.

**Pre-Pandemic Protocol Differences**

Prior to the GeoSmoking protocol, the study team designed, implemented, and collected data from a different version of this protocol (IRB 822815), which was approved by the University of Pennsylvania’s Institutional Review Board on September 22, 2015. This protocol largely overlapped with the GeoSmoking protocol in its main goals, but had several key differences, which make us expect that most papers resulting from this research will only examine data from the GeoSmoking protocol. The main distinction was that the previous protocol was used before the onset of the COVID-19 pandemic, and focused on in-person data collection rather than remote measures. All participants completed 3 in-person study sessions at the University of Pennsylvania. The second and third sessions (before and after the intervention period, respectively) included fMRI scans for all participants. Since all participants were scanned, recruitment targeted local populations who would be able to commute to Penn and would be eligible for fMRI scans. Recruitment was primarily conducted by the study team, not BuildClinical. More of the screening and eligibility confirmation steps were conducted at the first study session (for example, urine cotinine screening, urine drug screening, phone eligibility assessment) rather than the multi-step online screening done in the GeoSmoking version. The previous version also had additional categories in the fMRI task for some participants, additional EMA questions, and additional survey measures at the in-person appointments.

***Supplemental Figure 1****. Pre-pandemic recruitment flowchart.*

**
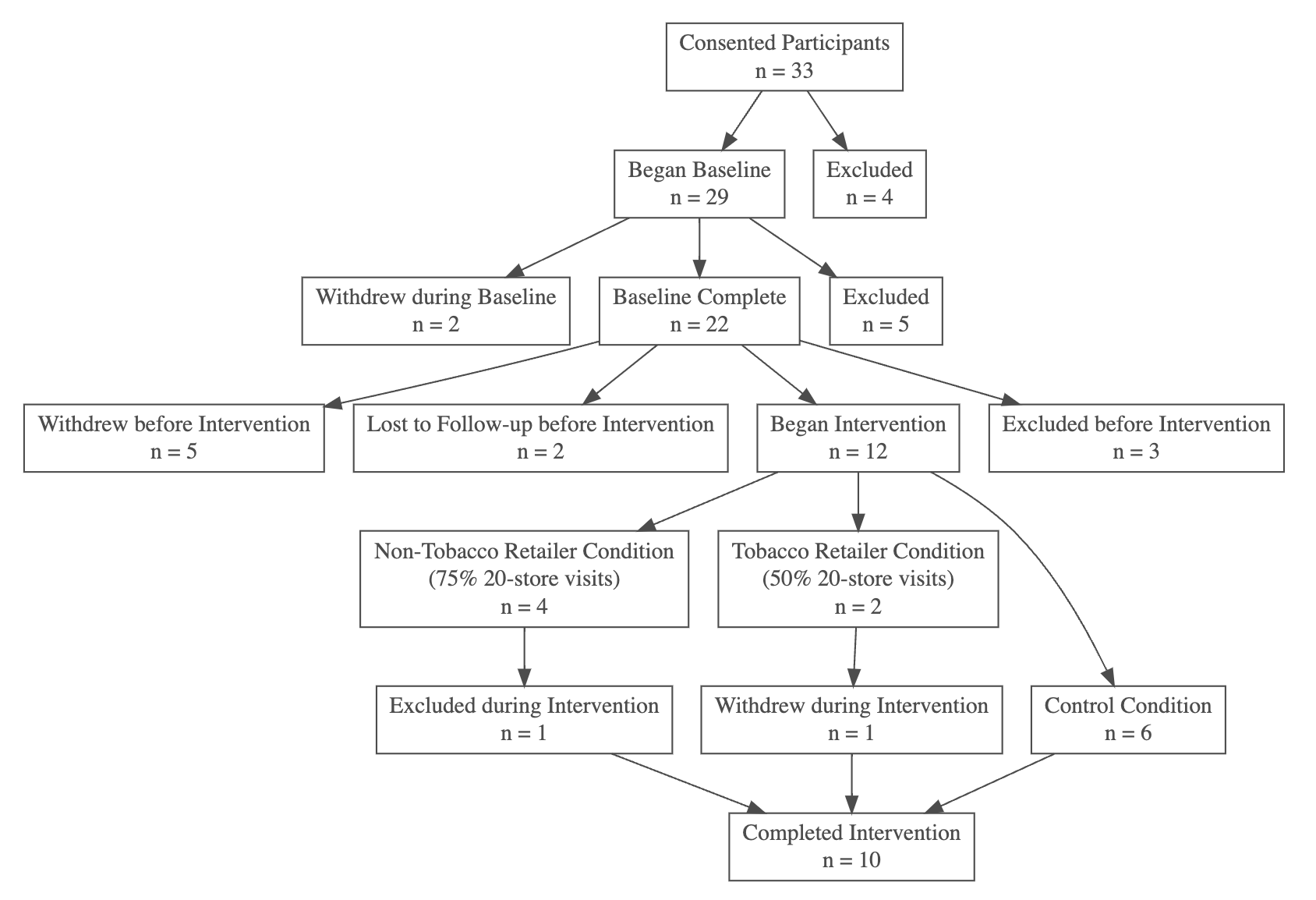
**

*Table S1. Demographics for participants in the tobacco retail condition, separated by those who completed baseline and began the intervention; completed the intervention period and submitted Online Session 3; and completed the intervention period with all 20 store visits. Values for age, MacArthur Subjective Social Status ladder, and cigarettes smoked per day are the mean and standard deviation (in parentheses).*


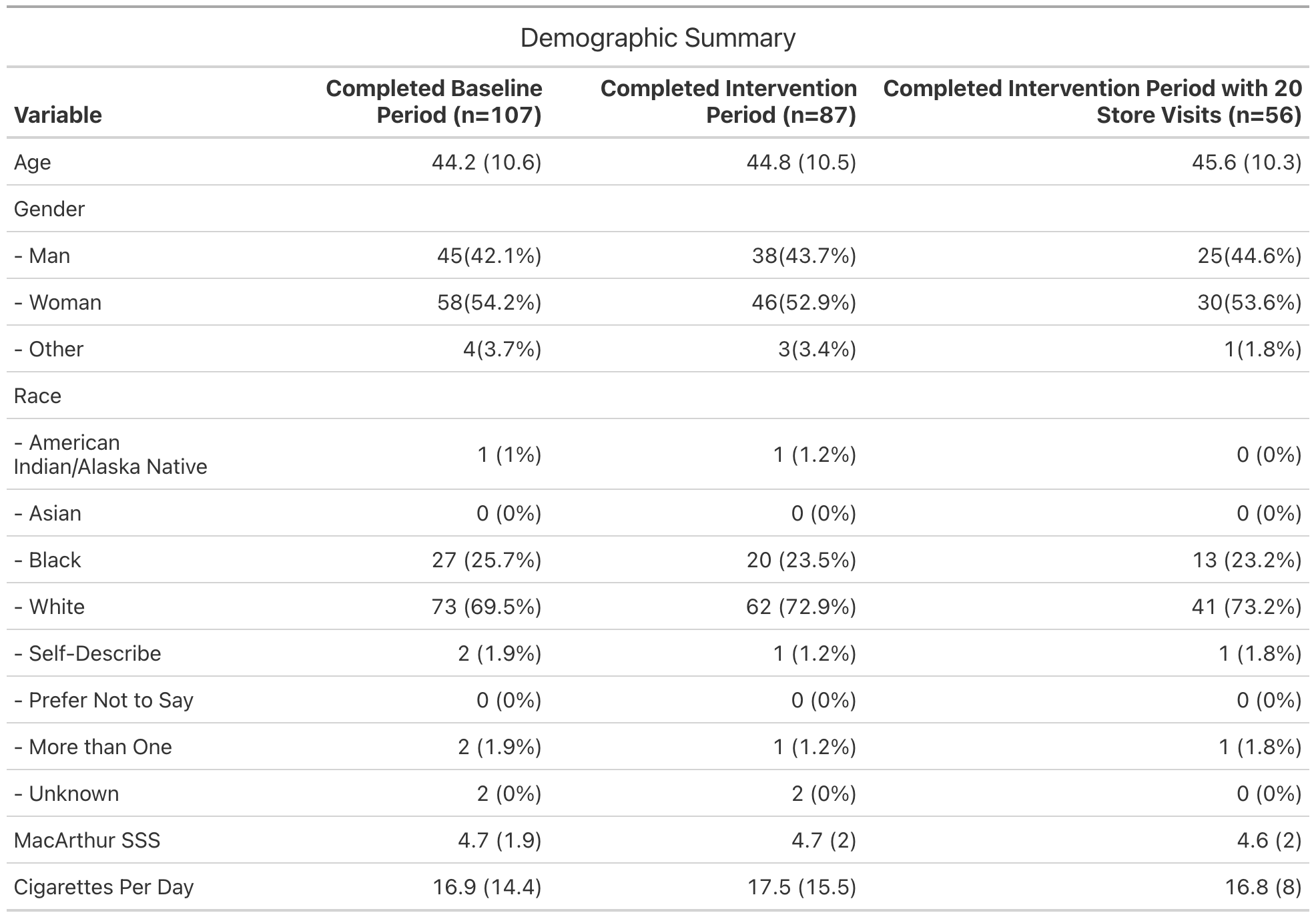


*Table S2. Demographics for participants in the non-tobacco retail condition, separated by those who completed baseline and began the intervention; completed the intervention period and submitted Online Session 3; and completed the intervention period with all 20 store visits. Values for age, MacArthur Subjective Social Status ladder, and cigarettes smoked per day are the mean and standard deviation (in parentheses).*

*
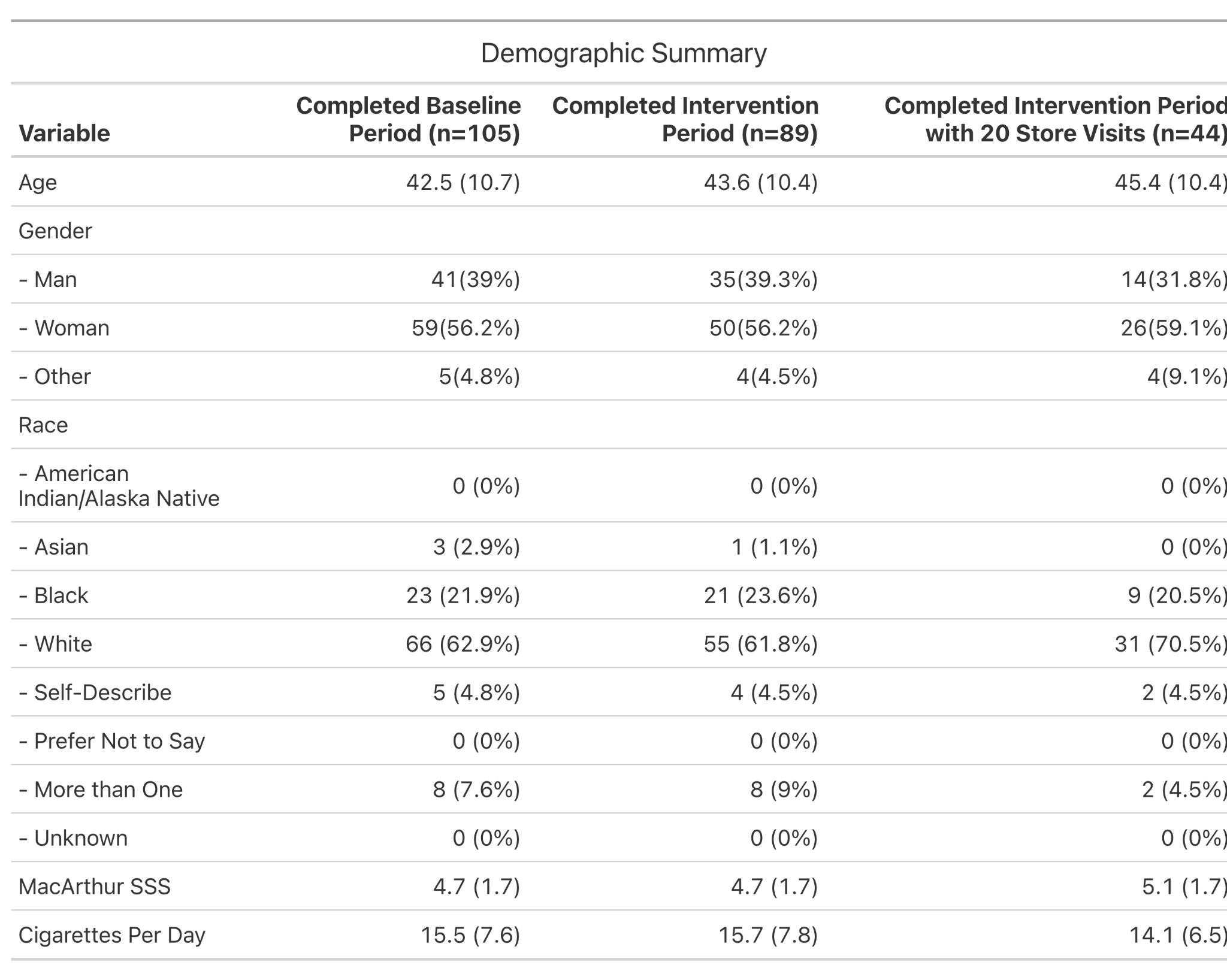
*

*Table S3. Demographics for participants in the control condition, separated by those who completed the baseline and began the intervention period, and those who completed the intervention period and submitted Online Session 3. Values for age, MacArthur Subjective Social Status ladder, and cigarettes smoked per day are the mean and standard deviation (in parentheses).*


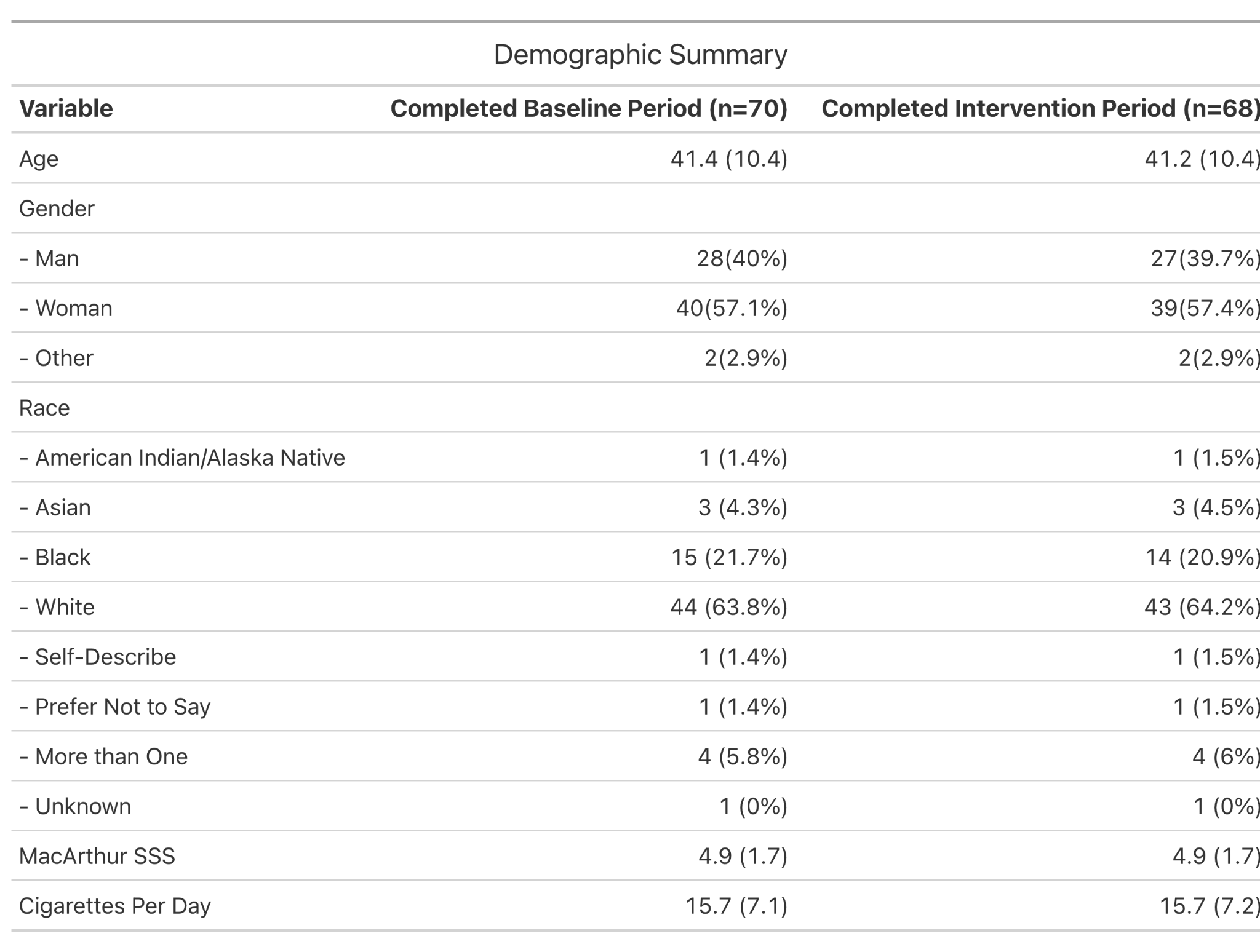

Supplement: Multimedia Appendix 1 [file resprot-v15-e89627-s001.docx]
